# Memory Performance for Everyday Motivational and Neutral Objects Is Dissociable from Attention

**DOI:** 10.3389/fnbeh.2017.00121

**Published:** 2017-06-26

**Authors:** Judith Schomaker, Bianca C. Wittmann

**Affiliations:** Department of Psychology, Justus Liebig UniversityGiessen, Germany

**Keywords:** memory, recollection, novelty, salience, attention

## Abstract

Episodic memory is typically better for items coupled with monetary reward or punishment during encoding. It is yet unclear whether memory is also enhanced for everyday objects with appetitive or aversive values learned through a lifetime of experience, and to what extent episodic memory enhancement for motivational and neutral items is attributable to attention. In a first experiment, we investigated attention to everyday motivational objects using eye-tracking during free-viewing and subsequently tested episodic memory using a remember/know procedure. Attention was directed more to aversive stimuli, as evidenced by longer viewing durations, whereas recollection was higher for both appetitive and aversive objects. In the second experiment, we manipulated the visual contrast of neutral objects through changes of contrast to further dissociate attention and memory encoding. While objects presented with high visual contrast were looked at longer, recollection was best for objects presented in unmodified, medium contrast. Generalized logistic mixed models on recollection performance showed that attention as measured by eye movements did not enhance subsequent memory, while motivational value (Experiment 1) and visual contrast (Experiment 2) had quadratic effects in opposite directions. Our findings suggest that an enhancement of incidental memory encoding for appetitive items can occur without an increase in attention and, vice versa, that enhanced attention towards salient neutral objects is not necessarily associated with memory improvement. Together, our results provide evidence for a double dissociation of attention and memory effects under certain conditions.

## Introduction

Anticipation and receipt of reward and punishment enhance long-term memory formation. A number of studies demonstrated memory enhancement after long retention intervals of >24 h, an effect that has been suggested to be mediated by dopaminergic modulation of hippocampal consolidation (Wittmann et al., 2005, 2008, 2011, 2013; Adcock et al., 2006; Krebs et al., 2009; Murty et al., 2012). Other studies reported reward-associated enhancement after short retention intervals (Callan and Schweighofer, 2008; Mather and Schoeke, 2011; Wolosin et al., 2012; Clewett and Mather, 2014), an effect that may be mediated by the effects of reward on attention, as it is well-established that attention strengthens memory encoding (for reviews see Chun and Turk-Browne, 2007; Uncapher and Wagner, 2009) and that motivationally salient stimuli draw attention (Raymond and O’Brien, 2009; Hickey et al., 2010, 2014; Hickey and van Zoest, 2012; Chelazzi et al., 2013; Hickey and Peelen, 2015). However, few studies have directly investigated how attention contributes to memory enhancement for motivational stimuli after short retention intervals, when dopaminergic modulation of consolidation is not yet completed. In addition, previous studies on motivational memory focused on the effects of explicit rewards and punishments delivered in an experimental setting, whereas the influence of prior experience with objects in the real world has not been addressed. Here, we set out to investigate whether memory is enhanced for everyday motivational objects, whether the effects of motivation on memory after short retention intervals are mediated by attention, and whether the memory effects of shifts of attention to neutral objects parallel the effects of motivation.

Throughout our lives we have acquired explicit knowledge about a wide range of objects (e.g., what they look like, how they feel, what they are used for); information that is represented in semantic memory (Patterson et al., 2007). In addition, we have learned to associate some objects with reward (e.g., a strawberry cake) and other objects with punishment (e.g., a thorny bush). These learned contingencies determine the expected value of interacting with an object and can be expressed as approach and avoidance behavior (Thorndike, 1911; Skinner, 1938; Huys et al., 2011; Aupperle et al., 2015). While objects thus have clear motivational properties, which could influence memory encoding in a similar way to explicitly delivered rewards, they can also elicit emotions (Ito et al., 1998; Lang et al., 2008). As emotional events are remembered better than neutral events (LaBar and Cabeza, 2006; Mather, 2007; Kensinger, 2009), it is important to control for effects of valence and (emotional) arousal when investigating motivation. Although emotion and motivation are interrelated, motivational drive and hedonic experience can be dissociated (for a review, see Berridge and Kringelbach, 2015). Another factor that may influence memory for natural stimuli and that therefore needs to be controlled when using real-world objects is recognizability, as it may be easier to remember stimuli that can be identified and named (for a review, see Saive et al., 2014).

In addition to its effects on long-term memory, motivationally salient information can draw attention (Raymond and O’Brien, 2009; Chelazzi et al., 2013). This is reflected in faster saccade latencies to face stimuli previously associated with reward (Rothkirch et al., 2013) and faster responses to targets presented at previously rewarded locations (Hickey et al., 2014). Attentional capture even occurs for task-irrelevant stimuli one has learned to associate with reward; target detection is slower when reward-associated stimuli are presented as distractors, even when they are visually equally salient compared to the other stimuli in the environment (Hickey et al., 2006, 2010; Anderson et al., 2011a,b; Awh et al., 2012; Hickey and van Zoest, 2012; Hickey and Peelen, 2015).

On one hand, memory can thus guide attention to information that has been proven to be valuable in the past (Awh et al., 2012; Chelazzi et al., 2013; Chun and Turk-Browne, 2007; Hickey et al., 2010, 2014, 2015; Hickey and van Zoest, 2012). On the other hand, attention is the mechanism by which information is selected for further processing and memory encoding (Chun and Turk-Browne, 2007; Seitz and Dinse, 2007; Carrasco, 2011). Accordingly, encoding of unattended information is impaired relative to attended information (Szymanski and MacLeod, 1996; MacDonald and MacLeod, 1998; Johnson and Zatorre, 2005), and memory performance is lower under conditions of divided attention (Kellogg et al., 1982; Park et al., 1989; Craik et al., 1996). This is consistent with findings that neural activity in attention-related brain regions predicts subsequent memory (Uncapher and Wagner, 2009; Kim, 2011). However, even though the two processes are strongly linked, they can be dissociated under certain conditions. A previous study investigating the effects of the emotional-motivational state of hunger on attention and memory found that attention effects did not predict enhanced memory for food items (Talmi et al., 2013).

While these studies show that improved memory encoding is not always associated with enhanced attention, other studies support the idea that attention is directed to novel information, which is preferentially encoded into long-term memory (Schomaker and Meeter, 2015). Attention to new rather than familiar information has been suggested to underlie so-called novelty preferences (Snyder et al., 2008). Humans have a preference to look at new compared to familiar information. Such novelty preferences are already observed in newborns (Snyder, 2007; Snyder et al., 2008). Interestingly, novelty preferences have been shown to disappear when the competing familiar stimuli are associated with emotionally salient information (Snyder, 2007). Emotional salience can thus override the novelty preferences, suggesting that an attentional mechanism underlies our preference to look at something new. The findings of Snyder (2007) suggest that novelty preferences should be affected by other factors influencing attention.

The current study included two experiments. In the first experiment, we investigated: (1) whether incidental episodic memory encoding is modulated by the intrinsic motivational value of everyday objects in the absence of external reinforcement; and (2) whether motivational memory effects after short retention intervals can be dissociated from attention and from effects of valence, arousal and recognizability. The analysis focused on recollection because of previous results on dopaminergic modulation of episodic memory processes in the hippocampus (Shohamy and Adcock, 2010; Lisman et al., 2011). We presented pictures of objects varying in motivational value, going from aversive (e.g., a fire extinguisher), through neutral (e.g., a stapler), to appetitive (e.g., a strawberry cake). We hypothesized that episodic memory would be enhanced for motivationally appetitive and aversive stimuli beyond possible contributions of attention and other stimulus-related factors such as valence, arousal and recognizability. The second experiment aimed to further specify the relationship between attention and memory. To assess the effect of shifts of attention on memory encoding, we manipulated the visual contrast of *neutral* everyday objects. We expected higher attention towards high-contrast stimuli and better memory for items receiving more attention. To induce attentional competition, we used an adaptation of a task typically used to investigate novelty preferences: the visual paired comparison (VPC) task. In the first phase of a VPC task, participants are familiarized with certain stimuli. In the subsequent VPC phase, the familiarized stimuli are presented together with new stimuli, often side by side. Novelty preferences can usually be observed during this phase as reflected by more and/or longer fixations on the novel compared to the previously familiarized stimuli. In this way, stimuli compete for attention during the VPC phase, and their different spatial location permits a quantification of attentional allocation per stimulus. This paradigm therefore allowed us to assess the role of attention in memory encoding for everyday motivational (Experiment 1) and contrast-modified (Experiment 2) objects. We subsequently tested recognition memory of the previously novel objects in a surprise memory task using the remember/know paradigm to assess recollection and familiarity (Tulving, 1985; Gardiner, 1988; Yonelinas and Jacoby’s, 1995).

## Methods

### Experiment 1

#### Participants

Ninteen volunteers with normal or corrected-to-normal vision participated in Experiment 1. Two participants were excluded because of calibration problems during eye tracking. Data analyses were performed on the data of the remaining participants (*n* = 17; age range 20–29 years; mean age = 23.35 years; 13 females). Participants either received course credit or a reimbursement of 8 € for their participation. This study was carried out in accordance with the recommendations of the ethics committee of the Department of Psychology and Sport Science at the Justus Liebig University Giessen, Germany. All subjects gave written informed consent in accordance with the Declaration of Helsinki.

#### Stimuli and Apparatus

Stimuli were pictures of objects taken from the Motivational Objects in Natural Scenes (MONS) database (Schomaker et al., unpublished data). This database includes >800 pictures of objects taken from natural scenes, each rated by 23–47 observers. Visual characteristics of the stimuli such as size and shape were similar across motivational categories, and a recent study using the MONS database demonstrated motivational effects on eye movements when controlling for visual aspects such as stimulus size and visual contrast (Schomaker et al., 2017). All objects and corresponding demographic and rating data are freely available at http://www.allpsych.uni-giessen.de/mons and will soon be uploaded to a permanent public repository. In the current study, a total of 216 objects was included, with 72 objects per motivational category. Motivational value as the average value of three scales: (1) Interaction: “How much would you like to interact with the object?”; (2) Own: “How much would you like to own the object?”; and (3) Approach/Avoid: “How much would you like to approach the object?”, each ranging from 1 (aversive) to 7 (appetitive) differed between categories: appetitive (mean motivational rating = 5.31, SD = 0.48; arousal rating = 4.13, SD = 0.61; valence rating = 5.01, SD = 0.42), neutral (mean motivational rating = 4.16; SD = 0.38; arousal rating = 3.68, SD = 0.35; valence rating = 4.11, SD = 3.74), and aversive (mean motivational rating = 3.13; SD = 0.61; arousal rating = 3.84, SD = 0.45; valence rating = 3.74, SD = 0.0.57). Objects were selected on basis of *ad hoc* assessment (intended motivation), but mean motivational ratings were confirmed to be different for the different motivational categories (aversive < neutral < appetitive: *p*s < 0.001). Differences were also observed for arousal (aversive > neutral, *p* = 0.015; appetitive > aversive, *p* < 0.001; appetitive > neutral, *p* < 0.001), and valence (aversive < neutral < appetitive, *p*s < 0.001). Since the motivational ratings were correlated with the valence ratings, we entered motivation, valence, arousal, and recognizability from the MONS database into generalized logistic mixed models to investigate their relative contributions to memory performance (see details below).

**Figure 1:**
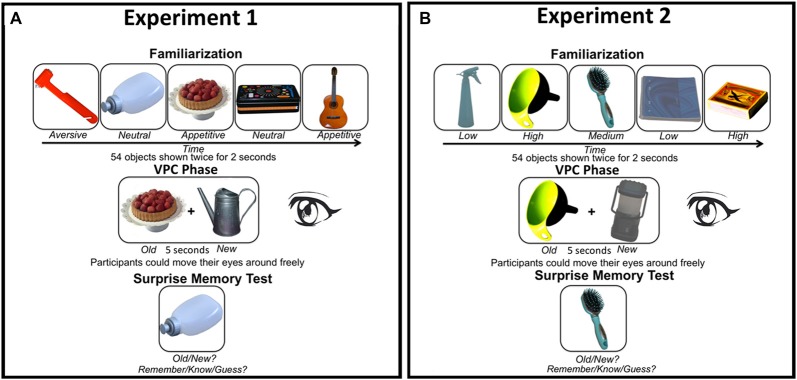
**(A)** Experimental design Experiment 1 and **(B)** Experiment 2. In both experiments we manipulated stimulus characteristics known to affect attention. In Experiment 1 the motivational value of the objects was varied (aversive, neutral, or appetitive), while contrast (low, medium, or high) varied between objects in Experiment 2. Both experiments started with a familiarization phase, followed by a visual paired comparison (VPC) phase in which a familiarized object was presented together with a new object (location randomized). Eye movements were only tracked during the VPC phase. Finally, participants performed an unannounced memory test in which they had to indicate whether the object on the screen was previously presented (“old”) or not (“new”). For “old” responses they also had to indicate whether they actually remembered seeing the object (“remember”), whether it was familiar (“know”), or whether they guessed (“guess”). Note that objects are depicted larger than in the actual experiments for demonstrational purposes (see “Methods” Section for image size details).

Example objects of each category can be found in Figure 1A. Images were presented on an LCD screen (1920 × 1080 pixels; 120 Hz refresh rate) and viewed at a distance of about 90 cm using a chin and head rest. Object stimuli were rescaled to fit in a 500 × 500 pixel box. This region was also used to define object fixations in the eye movement analyses. Each object covered about 10.75° × 10.75°. The experiment was created and presented using Open Sesame 2.9.0 (Mathôt et al., 2012).

Eighteen objects per motivational category (54 objects in total) were randomly chosen to be familiarized in the familiarization phase, and 18 different objects per category (54 in total) were randomly chosen to be presented as novel items in the VPC phase. Importantly, both the novel and familiar stimuli could vary in motivational value, such that any difference in viewing behavior between novel and familiar objects reflects memory of the familiarized object. Finally, the remaining 108 objects (36 per motivational category) were presented as lures in the memory test, intermingled with the 54 familiarized (“old objects with familiarization”) and 54 novel (“old objects without familiarization”) objects of the VPC phase.

Eye movements of the left eye were tracked using an Eyelink 1000 (SR Research Ltd., Kanata, ON, Canada) at a 1000 Hz sampling rate during the VPC phase of the experiment. A nine-point calibration was performed at the start of the experiment, and during the experiment between trials when the eyes were >60 pixels off at fixation. Saccade detection threshold was set at 30 degrees per second.

#### Design and Procedure

Figure 1A shows the experimental design and example stimuli in Experiment 1. The experiment was performed in a light-subdued room. Before participation, participants signed informed consent. Participants were instructed to look attentively at the stimuli presented. The experiment started with a familiarization phase in which trials started with the presentation of a fixation cross presented for a variable duration of 300–700 ms, followed by an object presented for 2000 ms. In this phase, 54 objects were presented in a random sequence. The same sequence was repeated once. The familiarization phase had a duration of approximately 5 min.

In the VPC phase, each trial started with a central fixation dot presented for at least 400–1000 ms, which had to be fixated to initiate the trial to ascertain that the eyes were in the middle of the screen at the onset of the object stimuli. After fixation, a new unfamiliarized and a familiar object were presented left and right of fixation (location randomized) for 5000 ms. Motivational value of both the familiar and novel stimuli varied in a counterbalanced fashion, such that each of the nine possible combinations of novelty status and motivational status occurred six times. Before this phase, participants were told they were allowed to move their eyes and let them wander freely during stimulus presentation. The VPC phase had an average duration of approximately 10 min.

Finally, in a surprise memory test the previously familiarized and new items of the VPC phase (from now on referred to as old with familiarization and old without familiarization, respectively) were presented intermingled with new lure objects. Participants were instructed to indicate whether they thought the object was presented at any time before in the experiment (“old”; press “x”) or whether they did not see the object before (“new”; press “m”). When an “old” response was given, participants had to further indicate whether they remembered (“remember”; press “y”) seeing the object, whether the object was only familiar (“know”; “v”), or whether they merely guessed (“guess”; press “m”). Each object was presented until a response was collected. The memory test phase was completed in approximately 10 min. Between the experimental phases, participants could take a short break. The entire experiment including instructions was completed in approximately 35–45 min.

#### Statistical Analyses

Fixations were classified as either on the novel or familiar (left or right) object during the VPC phase. For the eye movements, there were four measures of interest: (1) first fixation latency (in milliseconds); (2) first fixation type (quantified as the percentage of novel rather than familiar first fixations); (3) mean fixation duration (in milliseconds); and (4) total viewing duration (in milliseconds).

Novelty preferences were investigated using one-sample *t*-tests comparing the proportion of novel first fixations and the percentage of the novel total viewing duration to a test value of 0.5 (i.e., chance). To investigate the effects of motivation of both the novel and familiar stimulus on first fixation latency and mean fixation duration, 2*3 repeated measures ANOVA with novelty (novel; familiar) and motivation (appetitive; neutral; aversive) as factors were performed. The percentage of novel first fixations and percentage of novel total viewing duration were investigated with repeated measures ANOVAs with motivation (appetitive; neutral; aversive) as a factor.

In order to assess the contributions of motivation and attention to memory encoding, we analyzed memory performance for the novel objects (old objects without familiarization), which were only seen once during the VPC phase, when eye movements were recorded. The analysis was restricted to these objects because participants had seen the familiar objects twice before the VPC phase, making it impossible to to determine when memory encoding took place, thus precluding an analysis of eye movement effects, and leading to ceiling effects in the memory test. For completeness, we report the findings for the old objects with familiarization, which were very similar, in the Supplementary Materials. Since motivation could potentially have a non-linear effect on memory (both appetitive and aversive stimuli have previously been reported to enhance memory), we looked at both linear and quadratic effects. First, hits were defined as old objects without familiarization correctly classified as “old”, after excluding guesses, and false alarms as new items incorrectly identified as “old”, excluding guesses. Uncorrected hits per condition are reported in the Supplementary Material for completeness. To dissociate memory types, responses from the remember/know paradigm were used to derive recollection estimates (RE) and familiarity estimates (FE). Following Yonelinas and Jacoby’s (1995) procedure, RE were defined as the remember rate for hits corrected by subtracting false alarm rates (corrected remember responses). FE were calculated by first computing the “know” ratio for old stimuli, by dividing the know responses by 1 minus the remember responses for old stimuli (*F*_old_ = *K*_old_/(1 − *R*_old_)), and then subtracting the ratio of false alarm know responses for new stimuli (*F*_new_ = *K*_new_/(1 − *R*_new_)).

Both RE and FE were separately *z*-transformed before performing statistical analyses, to allow for comparisons. Memory performance was investigated by subjecting the *z*-transformed memory measures to a 2*3 repeated-measures ANOVA with memory type (RE; FE) and motivation (aversive; neutral; appetitive) as factors. Interaction effects between memory type and motivation were followed up with separate ANOVAs for RE and FE. A main effect of motivation was further investigated with planned contrasts by checking for quadratic effects. An interaction with memory type and motivation was followed up with separate ANOVAs for RE and FE (for more details see the “Results” Section). Finally, the effects were further investigated with *post hoc* *t*-tests comparing motivational categories separately for familiarity and recollection. For these *post hoc* comparisons per memory type the raw RE and FE measures were used. For all ANOVAs, Greenhouse-Geisser correction was performed when the sphericity assumption was violated.

##### Mixed effects logistic regression

To further investigate the factors contributing to recollection, we modeled the unique contributions of motivation, arousal and valence to remember responses using the ratings from the MONS database. For completeness, we also report models including both remember and know responses as a measure of overall recognition memory, which support the main findings, in the Supplementary Materials. As the effects of motivation and valence could potentially be non-linear (both appetitive and aversive stimuli may enhance memory compared to neutral stimuli), we also included squared motivation and valence regressors. In addition, we included total viewing duration in our main model, resulting in a full generalized logistic mixed model with eight fixed factors (motivation, arousal, valence, recognizability, total viewing duration, quadratic motivation, quadratic valence) and subject as a random effects factor. We also ran a model including random slopes for each subject, but this model did not differ from the model without random slopes (*p* = 0.99), therefore no random slopes were included in the models reported here. We also checked for collinearity by calculating variance inflation factors (vif). As we included both linear and quadratic versions for valence and motivation large vif values could be expected. Moreover, the valence and motivation scales in the MONS database are correlated. Indeed, vif values were high for motivation (vif = 67.5), quadratic motivation (vif = 73.0), valence (vif = 30.5) and quadratic valence (vif = 37.3), but not for arousal, total viewing time and recognizability (all vifs <1.3). To deal with collinearity, we centered the corresponding linear but not the quadratic factors around their means. This reduced the corresponding vif values to <3.1, which is well below the recommended maximum of 5 (Rogerson, 2001), or even 4 (Pan and Jackson, 2008). However, despite the reduced vif values, centered valence and centered motivation remained correlated (*r* = 0.69). As one of the goals of this study was to dissociate the effects of valence and motivation, we addressed this issue by first selecting only those items for which valence and motivation had an absolute difference ≥0.5. To investigate the separate effects of valence and motivation, we then selected two subsets of the images, one subset with neutral valence scores and one subset with neutral motivation scores (that is, ratings between 3.5 and 4.5; in line with the definition of neutral in the MONS database), thus reducing the correlation between the scales to *r* = 0.04 and *r* = −0.03, respectively. For the subset with neutral valence scores, 273 observations were included in a new model with the factors described above. In the subset with neutral motivation scores, 126 observations were included. Modeling was done in R version 3.2.5 (Bates et al., 2014) using the lme4 package. Models were compared using analyses of variance.

Attentional affects were investigated using four eye-movement measures. As we found behavioral effects of motivation and novelty on total viewing duration, we included this variable as a measure of attention in our main memory model. To further investigate the role of attention, additional models included first fixation latency and mean fixation duration of novel objects as predictors.

As we are interested in recollection, only “remember” responses were labeled as trials with successful encoding (assigning a 1), and all other responses as unsuccessful trials where no episodic encoding occurred (assigning a 0). Additional models on overall recognition memory that included both remember and know responses for old objects without familiarization as successful trials replicated the main effects of motivation, with the addition of trend effects for valence (Supplementary Materials, Table S1).

### Experiment 2

#### Participants

Seventeen volunteers with normal or corrected-to-normal vision participated in Experiment 2 (age range 20–27 years; mean age = 21.19 years; 14 females). Eye-tracking data of one participant were excluded because of technical difficulties. Participants who participated in Experiment 1 were excluded from participation. Participants either received course credit or a reimbursement of 8 €. The experiment was approved by the local ethics committee of the Department of Psychology and Sport Science at the Justus Liebig University Giessen, Germany, and all participants signed informed consent.

#### Stimuli and Apparatus

**Figure 2:**
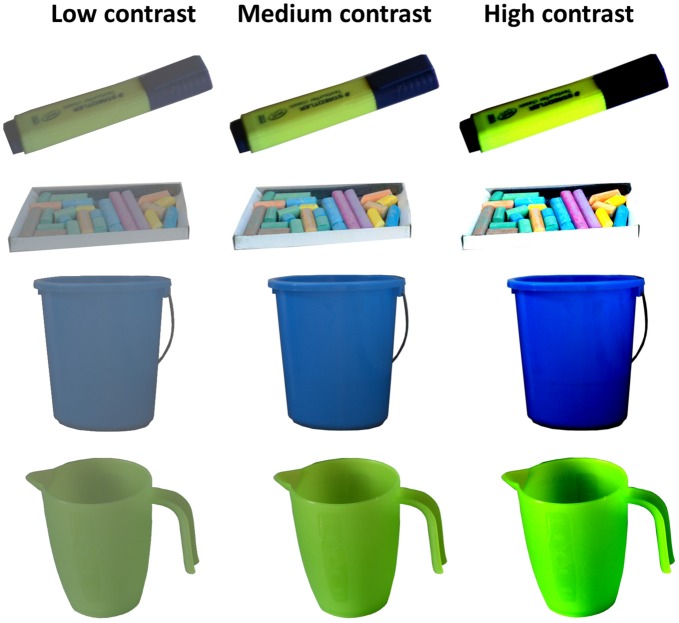
Example objects with contrast manipulation. Each individual object was presented at the same contrast level throughout the experiment, counterbalanced across subjects.

The apparatus was the same as in Experiment 1. The stimuli (216 in total) were taken from the same MONS database, but now only ground truth neutral objects were included (that is, objects *ad hoc* estimated to have neutral motivational value before the database ratings were available). Average neutral value was confirmed by the mean motivational rating = 4.25 (on a 1- to 7-point scale), SD = 0.65; arousal rating = 3.60, SD = 0.35; valence rating = 4.14, SD = 0.48. The contrast of all objects was increased by 64%, decreased by 64% or not manipulated, resulting in three versions of each image with low, medium (normal), and high contrast (for examples see Figure 2).

#### Design and Procedure

The design and procedure were the same as in Experiment 1, with stimuli as the only change. Instead of objects from three motivational categories, neutral objects were presented at three levels of visual contrast. Examples can be found in Figure 1B. One third of the objects was presented with low, one third with normal and one third with high contrast (i.e., 6 trials for each stimulus combination). Contrast was not manipulated for objects within the experiment, such that the same contrast was used for a certain image in the familiarization, VPC, and memory test phase. Contrast varied between subjects in a random fashion (e.g., the colander presented in low contrast for one participant could be presented in high contrast for another participant).

#### Statistical Analyses

The same statistical tests were performed as in Experiment 1 with the factor contrast (low, medium, high) instead of the factor motivation. We expected a linear effect of contrast on eye movements, but possibly quadratic effects on memory since the contrast manipulation potentially changed the recognizability of the stimuli and could thereby also negatively affect memory. Effects of visual contrast were further investigated with planned contrasts (low vs. medium; medium vs. high; low vs. high).

##### Mixed effects logistic regression

We ran the same generalized logistic mixed models as for Experiment 1, with the exception that visual contrast was now added as a regressor. To label visual contrast, low contrast objects were given a value of 1, medium contrast objects of 2, and high contrast objects of 3. Contrast may affect memory for the visually manipulated types of stimuli: memory may be affected for both low and high contrast compared medium contrast stimuli, therefore we also included a squared contrast regressor.

## Results

### Experiment 1

#### Eye Movements

**Figure 3:**
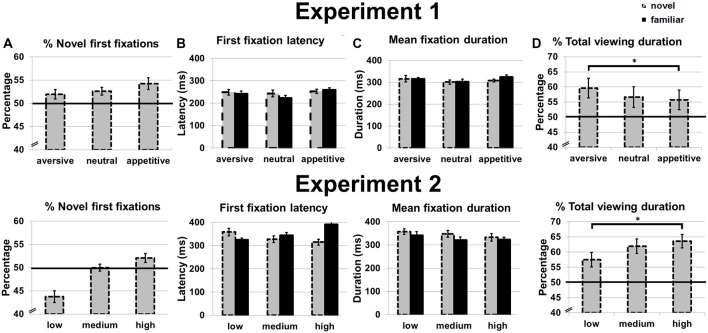
Eye movement measures in Experiment 1 (top) and Experiment 2 (bottom). **(A)** Percentage of first fixations on novel stimuli. **(B)** First fixation latency for novel and familiar stimuli. **(C)** Mean fixation duration for novel and familiar stimuli. **(D)** Percentage of total viewing duration on novel stimuli. Error bars reflect standard error of the mean. *p < 0.05.

Mean proportion of first fixations, first fixation latencies, mean fixation duration and proportion of total novel viewing duration for familiar and novel objects can be found in Figure 3. A novelty preference was found for the total viewing duration; the proportion of time the participants viewed the novel rather than the familiar stimuli was higher than chance (0.5), *t*_(16)_ = 2.73, *p* = 0.015. Also a first fixation was more likely than chance to be on a novel stimulus, *t*_(16)_ = 2.89, *p* = 0.011.

The repeated measures ANOVAs showed no main effect of motivation on the percentage of novel first fixations (*p* = 0.759; Figure 3A). For first fixation latency, there were no main effects of motivation (*p* = 0.193) or novelty (*p* = 0.537) and no interaction (*p* = 0.682; Figure 3B). For mean fixation duration, there were also no main effects of novelty (that is, no novelty preference in dwell time; *p* = 0.62) or motivation (*p* = 0.332) and no interaction (*p* = 0.730; Figure 3C).

While no main effect of motivation was observed on the percentage of total viewing duration for novels (*p* = 0.136), a linear effect was found: total viewing duration increased from appetitive to aversive objects *F*_(1,16)_ = 5.11, *p* = 0.036, *η*^2^ = 0.22 (Figure 3D). *Post hoc t*-tests confirmed a longer total viewing duration for aversive compared to appetitive objects, *t*_(16)_ = 2.29, *p* = 0.036, while duration between aversive and neutral, and neutral and appetitive objects did not differ (*p* = 0.210 and *p* = 0.647, respectively). Since no effects were observed on mean fixation duration, the effects observed on total viewing duration were caused by a higher number of (re)visitations to the novel stimuli.

#### Memory

**Figure 4:**
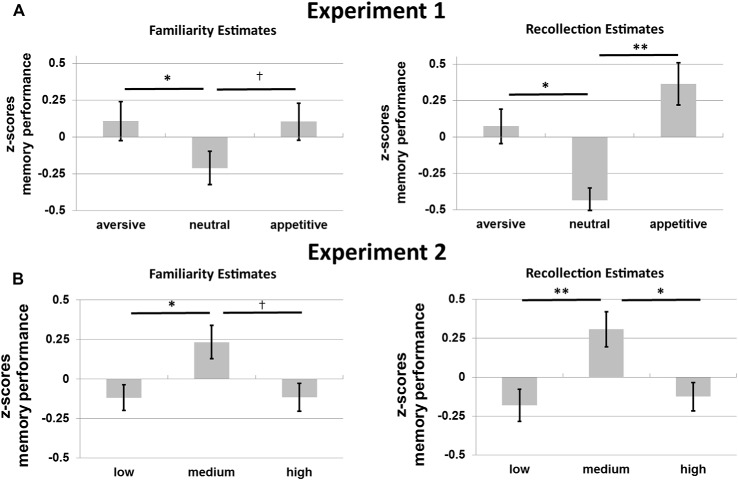
Memory performance in *z*-scores for **(A)** Experiment 1 and **(B)** Experiment 2. Familiarity estimates (FE; left) and recollection estimates (RE; right) for old objects without familiarization for the three motivational categories (aversive, neutral and appetitive) in Experiment 1 and for the three levels of visual contrast (low, medium and high) in Experiment 2. Error bars reflect standard errors of the mean. ^†^*p* < 0.10; *p < 0.05; **p < 0.01.

Figure 4A shows the average memory performance as defined by the FE and RE for the old objects without familiarization in Experiment 1. Memory performance for these objects was investigated with a 2*3 repeated-measures ANOVA with memory type (RE, FE), and motivation (appetitive, neutral, aversive) as factors. There was no main effect of memory type (*p* = 0.748), but there was a main effect of motivation on memory, *F*_(2,32)_ = 8.56, *p* = 0.001, *η*^2^ = 0.35, motivation exhibiting a quadratic effect of motivation, *F*_(1,16)_ = 32.72, *p* < 0.001, *η*^2^ = 0.67: memory was improved for the motivational compared to the neutral objects. No interaction was observed between memory type and motivation (*p* = 0.564).

Planned *post hoc* ANOVAs per memory type with motivation as a factor were performed to further specify the effects of motivation. The effects of motivation on recollection were quadratic: participants remembered more aversive and appetitive compared to neutral stimuli, *F*_(1,16)_ = 24.91, *p* < 0.001, *η*^2^ = 0.61 (linear effect, *p* = 0.340). For familiarity a similar quadratic effect of motivation was found, *F*_(1,16)_ = 9.36, *p* = 0.007, *η*^2^ = 0.37 (linear effect, *p* = 0.799).

*Post hoc t*-tests revealed higher recollection for aversive and appetitive compared to neutral objects, *t*_(16)_ = 2.69, *p* = 0.016 and *t*_(16)_ = 3.88, *p* = 0.001, respectively. Recollection did not differ between aversive and appetitive objects (*p* = 0.340). Familiarity was higher for aversive compared to neutral objects, *t*_(16)_ = 2.32, *p* = 0.034. Familiarity did not differ between aversive and appetitive nor between appetitive and neutral objects (*p* = 0.799 and *p* = 0.068).

The main generalized logistic mixed model showed that motivation and squared motivation contributed to recollection performance (motivation: *p* < 0.001; motivation^2^: *p* < 0.001; see Table 1 for all model results). Except for recognizability, which showed a trend effect (*p* = 0.049), all other regressors (valence, valence^2^, arousal, recognizability and total viewing duration) did not significantly contribute (*p* > 0.124). The model predicted 67.3% of the data accurately (*p* < 0.001).

**Table 1:** Generalized logistic mixed model results on remember responses to old objects without familiarization, separately for Experiment 1 and 2.

	Experiment 1	Experiment 2
Observations	1026 for 19 subjects	918 for 17 subjects
Motivation	***E* = −2.01, SE = 0.52, *z* = 3.87, *p* < 0.001**	*E* = −0.61, SE = 1.60, *z* = −0.39, *p* = 0.699
Quadratic motivation	***E* = 0.24, SE = 0.07, *z* = 3.76, *p* < 0.001**	*E* = 0.03, SE = 0.19, *z* = 0.19, *p* = 0.846
Valence	*E* = 0.10, SE = 0.48, *z* = 0.19, *p* = 0.835	*E* = 3.23, SE = 2.79, *z* = 1.16, *p* = 0.246
Quadratic valence	*E* = −0.01, SE = 0.06, *z* = −0.17, *p* = 0.853	*E* = −0.33, SE = 0.33, *z* = −1.00, *p* = 0.320
Arousal	*E* = 0.22, SE = 0.15, *z* = 1.45, *p* = 0.124	***E* = 0.47, SE = 0.22, *z* = 2.13, *p* = 0.033**
Recognizability	***E* = 0.26, SE = 0.13, *z* = 1.98, *p* = 0.048**	***E* = 0.33, SE = 0.14, *z* = 2.25, *p* = 0.025**
Total viewing duration	*E* = 0.28, SE = 0.23, *z* = 1.22, *p* = 0.222	*E* = 0.44, SE = 0.36, *z* = 1.24, *p* = 0.214
Visual contrast	–	***E* = 1.91, SE = 0.62, *z* = 3.10, *p* = 0.002**
Quadratic visual contrast	–	***E* = −0.49, SE = 0.15, *z* = −3.20, *p* = 0.001**

*Significant effects (p < 0.05) are printed in bold. E, Estimate; SE, standard error*.

We found that valence and motivational ratings were correlated in our data set, *r* = 0.69, causing collinearity in the logistic mixed model. To further investigate whether valence and motivation could be dissociated, we made a *post hoc* selection including only objects with a minimum difference between valence and motivation, and neutral ratings for valence (neutral valence model) or neutral ratings for motivation (neutral motivation model; see “Methods” Section for more details on this *post hoc* selection). In both cases correlations between valence and motivation were reduced to non-significant levels (*r* = 0.04 and *r* = −0.03, respectively). Results of the neutral valence model confirm a significant motivational effect (both linear and quadratic: *p* < 0.001; Table 2). In addition, we found a contribution of arousal (*p* < 0.01). For the neutral motivation model, none of the regressors significantly predicted recollection (all *p*s > 0.139; Table 2).

**Table 2:** Generalized logistic mixed model results on remember responses to old objects without familiarization for Experiment 1, separately for a neutral valence model and a neutral motivation model.

	Neutral valence model	Neutral motivation model
Observations	273 for 19 subjects	126 for 17 subjects
Motivation	***E* = −7.14, SE = 2.06, *z* = −3.47, *p* < 0.001**	*E* = 57.25, SE = 38.72, *z* = 1.48, *p* = 0.139
Quadratic motivation	***E* = 0.94, SE = 0.28, *z* = 3.42, *p* < 0.001**	*E* = −6.96, SE = 4.80, *z* = −1.45, *p* = 0.147
Valence	*E* = 18.60, SE = 16.69, *z* = 1.11, *p* = 0.265	*E* = 1.24, SE = 0.96, *z* = −1.29, *p* = 0.198
Quadratic valence	*E* = −2.28, SE = 2.09, *z* = −1.09, *p* = 0.276	*E* = 0.21, SE = 0.17, *z* = −1.24, *p* = 0.216
Arousal	***E* = −1.15, SE = 0.44, *z* = −2.58, *p* = 0.010**	*E* = −0.16, SE = 1.01, *z* = −0.16, *p* = 0.871
Recognizability	*E* = 0.09, SE = 0.25, *z* = 0.39, *p* = 0.700	*E* = 0.40, SE = 0.55, *z* = 0.73, *p* = 0.464
Total viewing duration	*E* = 0.25, SE = 0.51, *z* = 0.49, *p* = 0.623	*E* = −0.15, SE = 0.90, *z* = −0.17, *p* = 0.867

*Significant effects (p < 0.05) are printed in bold. E, Estimate; SE, standard error*.

The attentional contribution to memory performance was further investigated with additional models including either first fixation latency (Table 3) or mean novel fixation duration (Table 4) as predictors instead of total viewing duration, but these variables did not predict memory performance (*p* = 0.122 and *p* = 0.109, respectively. Models including remember plus know responses also did not reveal any attention effects (see Supplementary Materials; model including first fixation latency: Supplementary Table S2; model including mean novel fixation duration: Supplementary Table S3).

**Table 3:** Generalized logistic mixed model results on remember responses to old objects without familiarization including first fixation latency as a predictor, separately for Experiment 1 and 2.

	Experiment 1	Experiment 2
Observations	1026 for 19 subjects	918 for 17 subjects
Motivation	*E* = 0.02, SE = 0.13, *z* = 0.18, *p* = 0.859	*E* = −0.30, SE = 0.17, *z* = −1.78, *p* = 0.075
Quadratic motivation	***E* = 0.29, SE = 0.07, *z* = 4.07, *p* < 0.001**	*E* = 0.05, SE = 0.19, *z* = 0.28, *p* = 0.783
Valence	*E* = 0.04, SE = 0.18, *z* = 0.21, *p* = 0.837	***E* = 0.48, SE = 0.21, *z* = 2.30, *p* = 0.022**
Quadratic valence	*E* = −0.06, SE = 0.08, *z* = −0.73, *p* = 0.466	*E* = −0.38, SE = 0.34, *z* = −1.13, *p* = 0.257
Arousal	*E* = 0.26, SE = 0.15, *z* = 1.70, *p* = 0.089	***E* = 0.47, SE = 0.22, *z* = 2.16, *p* = 0.031**
Recognizability	*E* = 0.26, SE = 0.13, *z* = 1.95, *p* = 0.051	***E* = 0.31, SE = 0.15, *z* = 2.15, *p* = 0.032**
First fixation latency	*E* < 0.01, SE < 0.01, *z* = −1.55, *p* = 0.122	*E* < −0.01, SE < 0.01, *z* = −1.00, *p* = 0.316
Visual contrast	–	***E* = 1.92, SE = 0.62, *z* = 3.10, *p* = 0.001**
Quadratic visual contrast	–	***E* = −0.49, SE = 0.15, *z* = −3.18, *p* = 0.002**

*Significant effects (p < 0.05) are printed in bold. E, Estimate; SE, standard error*.

**Table 4:** Generalized logistic mixed model results on remember responses to old objects without familiarization including mean novel fixation duration as a predictor, separately for Experiment 1 and 2.

	Experiment 1	Experiment 2
Observations	1026 for 19 subjects	918 for 17 subjects
Motivation	*E* = 0.05, SE = 0.13, *z* = 0.35, *p* = 0.725	***E* = −0.34, SE = 0.17, *z* = −2.02, *p* = 0.043**
Quadratic motivation	***E* = 0.30, SE = 0.07, *z* = 4.21, *p* < 0.001**	*E* = 0.05, SE = 0.19, *z* = 0.27, *p* = 0.789
Valence	*E* = 0.05, SE = 0.18, *z* = 0.28, *p* = 0.783	***E* = 0.52, SE = 0.21, *z* = 2.50, *p* = 0.012**
Quadratic valence	*E* = −0.05, SE = 0.08, *z* = −0.64, *p* = 0.522	*E* = −0.44, SE = 0.34, *z* = −1.29, *p* = 0.198
Arousal	*E* = 0.22, SE = 0.15, *z* = 1.42, *p* = 0.155	***E* = 0.45, SE = 0.22, *z* = 2.02, *p* = 0.043**
Recognizability	*E* = 0.25, SE = 0.13, *z* = 1.84, *p* = 0.066	***E* = 0.32, SE = 0.14, *z* = 2.20, *p* = 0.028**
Mean novel fixation	E < −0.01, SE < 0.01, *z* = −1.60, *p* = 0.109	***E* < −0.01, SE < 0.01, *z* = −2.46, *p* = 0.014**
Visual contrast	–	***E* = 2.07, SE = 0.62, *z* = 3.32, *p* < 0.001**
Quadratic visual contrast	–	***E* = −0.52, SE = 0.15, *z* = −3.42, *p* < 0.001**

*Significant effects (p < 0.05) are printed in bold. E, Estimate; SE, standard error*.

### Experiment 2

Results from Experiment 1 suggest that the effects of motivational value on attention and memory are dissociable. To further investigate the role of attention in memory formation irrespective of motivation in Experiment 2, we used the same paradigm, but manipulated the visual contrast (low, medium, or high) of objects with neutral motivational value (Figure 1B).

#### Eye Movements

Mean proportion of first fixations, first fixation latencies, mean fixation duration and proportion of total novel viewing duration for familiar and novel objects can be found in Figure 3. A novelty preference was found for the total viewing duration; the novel images were viewed longer than chance, *t*_(15)_ = 5.24, *p* < 0.001. Novel stimuli were not more likely to be fixated first than chance (*p* = 0.227), and also the first fixation latency was similar for novel and familiar stimuli (*p* = 0.382).

There was no main effect of contrast on the percentage of novel first fixations (*p* = 0.190; Figure 3A) in a one-way ANOVA, although there was a statistical trend for a linear effect with more novel first fixations for objects presented in high vs. lower contrast, *F*_(1,15)_ = 3.22, *p* = 0.093, *η*^2^ = 0.18.

We next carried out 2 × 3 ANOVAs with the factors novelty and contrast on first fixation latency and mean fixation duration. No main effects of novelty or contrast were observed for first fixation latency (*p* = 0.342 and *p* = 0.676 respectively), but the two factors interacted, *F*_(2,30)_ = 5.75, *p* = 0.008, *η*^2^ = 0.28 (Figure 3B). This interaction effect was linear, with the first fixation latency on novel objects decreasing for increasing contrast of the novel, and the first fixation latency on familiar objects increasing with increasing contrast of the novel object, *F*_(1,15)_ = 9.83, *p* = 0.007, *η*^2^ = 0.40. For mean fixation duration, there was a main effect of novelty, *F*_(1,15)_ = 4.79, *p* = 0.045, *η*^2^ = 0.24. *Post hoc t*-tests showed that this effect was not very robust; only a trend for longer mean viewing duration for novels compared to familiar objects presented with medium contrast was observed, *t*_(15)_ = 1.97, *p* = 0.068 (for low and high contrast *ps* > 0.263). No main effect of contrast (*p* = 0.109) and no interaction were observed (*p* = 0.758; Figure 3C).

There was a main effect of contrast on the percentage of total viewing duration for novels, *F*_(2,30)_ = 5.59, *p* = 0.007, *η*^2^ = 0.28, as also evidenced by a linear effect with a higher percentage of total viewing duration for novel objects presented in high compared to lower contrast, *F*_(1,15)_ = 11.44, *p* = 0.004, *η*^2^ = 0.43 (Figure 3D). *Post hoc t*-tests showed that indeed the high contrast novel objects were looked at longer than the low contrast novel objects, *t*_(15)_ = 3.38, *p* = 0.004, and a trend effect for longer viewing durations for novel objects presented in medium vs. low contrast, *t*_(15)_ = 2.09, *p* = 0.054. Total viewing duration did not differ for objects presented in medium and high contrast (*p* = 0.267).

#### Memory

Figure 4B shows RE and FE for old objects without familiarization for Experiment 2. A 2 × 3 repeated-measures ANOVA with the factors memory type and contrast revealed a main effect of visual contrast on memory performance for these objects, *F*_(2,30)_ = 8.74, *p* = 0.001, *η*^2^ = 0.37, with a significant quadratic effect, *F*_(2,30)_ = 16.49, *p* = 0.001, *η*^2^ = 0.52, but no linear effect (*p* = 0.793). No interaction between memory type and contrast was found (*p* = 0.803).

These results were followed up with separate repeated-measures ANOVAs per memory type (RE and FE; with visual contrast as a factor). Results from these additional tests revealed a quadratic effect of contrast on recollection, *F*_(1,15)_ = 11.82, *p* = 0.004, *η*^2^ = 0.44, with worse recollection for objects presented in low or high, compared to medium contrast (no linear effect: *p* = 0.744). For familiarity a similar quadratic effect was observed, *F*_(1,15)_ = 5.78, *p* = 0.030, *η*^2^ = 0.28 (no linear effect: *p* = 0.984).

Planned *post hoc t*-tests revealed higher recollection for objects presented with medium vs. low, *t*_(15)_ = 3.10, *p* = 0.007, and medium vs. high contrast, *t*_(15)_ = 2.73, *p* = 0.015, and no differences between low and high contrast (*p* = 0.744). Familiarity was only higher for medium compared to low contrast objects, *t*_(15)_ = 2.44, *p* = 0.027. No differences between the other contrast levels were observed (low vs. high: *p* = 0.984; medium vs. high: *p* = 0.056, but with a trend for higher familiarity for medium vs. high contrast).

The generalized logistic mixed model confirmed that visual contrast influenced recollection performance (visual contrast: *p* = 0.002 and visual contrast^2^: *p* = 0.001). In addition, arousal and recognizability contributed to recollection (*p*s < 0.05; see Table 1 for detailed model results). All other regressors (motivation, motivation^2^, valence, valence^2^ and total viewing duration) had no significant contribution (*p*s > 0.213). The model including remember plus know responses only revealed effects of visual contrast (see Supplementary Materials: Table S1).

The model including first fixation latency also did not reveal an attentional effect (*p* = 0.316; Table 3). The model including mean novel fixation duration indicated that this variable predicted memory performance (*p* = 0.014; Table 4), but this was a negative effect, with longer mean fixation duration related to lower memory performance in line with the finding that memory was lower for high-contrast objects despite longer viewing times for these objects. Models including remember plus know responses did not reveal any attention effects (see Supplementary Materials; model including first fixation latency: Supplementary Table S2; model including mean novel fixation duration: Supplementary Table S3).

## Discussion

In the present study, we used a task typically used to study novelty preferences to investigate the role of attention in incidental memory encoding of motivationally and visually salient stimuli. In the first experiment we varied the motivational value of the objects presented, while in the second experiment we varied the visual contrast independently of motivational value, which was kept neutral. Attention and memory effects were dissociable in both experiments, with enhanced episodic memory for appetitive and aversive motivational objects despite longer viewing of only the aversive objects, and decreased episodic memory for objects with high and low contrast despite longer viewing of the high-contrast objects. Our results thus indicate that the effects of motivation on memory encoding are separable from its effects on attention, and the opposite, that attentional allocation is not sufficient for memory enhancement to occur.

In both experiments, new images were looked at longer than familiar images, consistent with typically observed novelty preferences (Snyder, 2007). Viewing duration of novel objects was longer for aversive objects and objects presented with high contrast, but no interaction between object novelty and motivation or contrast on viewing duration was found, suggesting separate, additive effects of motivation, contrast and novelty on attention.

The effects of motivation on attention were dissociable from its effects on memory performance. Although novel aversive objects were looked at longer than appetitive objects, episodic memory was equally improved for appetitive and aversive compared to neutral objects. That viewing duration was not a main contributor to memory is also supported by our modeling results: in both experiments, no significant contribution of total viewing duration on memory for the novel stimuli was found.

In the present study, the motivational value of everyday objects thus promoted incidental memory encoding through a mechanism other than attention. Previous studies found co-activation of dopaminergic areas and the hippocampus during encoding of items associated with extrinsic rewards and punishments (Wittmann et al., 2005, 2013; Adcock et al., 2006; Callan and Schweighofer, 2008). It is possible that this mechanism could also underlie the effects of intrinsic motivational associations that are learned through experience in the real world. However, dopamine has been found to specifically affect hippocampal memory consolidation, leading to memory enhancement after long but not short retention intervals (for a review, see Lisman et al., 2011).

Another possibility is that motivational memory enhancement was mediated by arousal. The appetitive and aversive objects in this study received higher arousal ratings than the neutral objects, and arousal has been found to enhance memory (e.g., Hamann et al., 1999; Dolcos et al., 2004; Mather and Sutherland, 2009). Therefore, we included arousal ratings in the generalized logistic mixed models. In Experiment 1, arousal did not contribute to recollection in the full model and in the neutral motivation model, but there was a significant effect in the neutral valence model. Arousal also contributed significantly in Experiment 2. The arousal effect on memory has been suggested to reflect enhanced attention to and consolidation of arousing items (LaBar and Cabeza, 2006; Mather, 2007; Kensinger, 2009). While the current results thus confirm a role of arousal in memory, the significant contribution of motivation to recollection indicates that intrinsic motivational value improves episodic memory encoding beyond its arousing properties. For emotional items, factors such as semantic relatedness and perceptual vividness also play a role in memory formation (Talmi et al., 2007; Todd et al., 2012, 2013; Talmi, 2013). Future studies will be needed to investigate the contribution of these factors to motivational memory enhancement after short retention intervals.

Motivational value depends on the outcome of interacting with a certain stimulus. Emotion can highlight which stimuli are relevant in order to guide behavior to obtain rewards and avoid punishment (Frijda, 2007). While emotional valence corresponds to the hedonic experience (“liking”) associated with a stimulus, motivation refers to the behavioral drive (“wanting”) elicited by it (Berridge and Robinson, 2003). In many studies, however, valence and motivation are conflated (Berridge et al., 2009), and since both motivational value and emotion have a range of effects on cognition and behavior it is often difficult to dissociate the two. In the current study, however, we did not find a contribution of valence in our recollection models when total viewing duration was taken into account. This finding was further supported by additional analyses on subsets of the data, which confirmed the effects of motivation on recollection when restricting the analysis to emotionally neutral objects, whereas no effects of valence were found when including only motivationally neutral objects. Note however, that because these additional analyses relied on subsets of the data, the absence of emotional effects should be cautiously interpreted. A reason for our finding of motivational but no emotional effects on memory could be that the MONS database was designed to elicit stronger motivational than emotional effects. In line with this aim, the ratings on the valence scale are closer to neutral than the ratings on the motivational scales, supporting the idea that the contributions of motivation in our study were more substantial than those of valence. Previous results have shown that emotional memory enhancement for positive pictures is mediated by attention, whereas emotional memory for negative pictures occurs independently of attention (Talmi et al., 2007). Since less attention was allocated to appetitive objects compared to aversive objects in the current study, enhanced memory for the appetitive items cannot be explained by effects of positive emotions, which would be expected to be mediated by attention. This interpretation is also in line with the recent finding that hungry participants’ memory for appetitive food images is not mediated by attention (Talmi et al., 2013), as food has high motivational value in a state of hunger. One limitation of the present study is that such differences in internal states were not controlled and could potentially have affected our findings. Nevertheless, we do find overall effects of motivation on our dependent variables (eye movements/attention and subsequent memory), suggesting that state-dependent effects were minor.

In the second experiment, memory effects were also dissociable from attention. While objects presented in high vs. low visual contrast were looked at longer, memory performance was better for objects presented in medium contrast. Contrast determines the color and brightness of an image, and both of these local object properties have been suggested to play a role in object recognition (Roth and Winter, 2008). Our contrast manipulation could therefore have impaired object recognition for the objects presented in low or high contrast, making it more difficult to label them, impairing memory encoding as a result (Saive et al., 2014). This interpretation is supported by the finding from the models for both experiments that recognizability positively contributed to episodic memory: objects rated as more recognizable in the MONS database were remembered better. Independent of the mechanism underlying the observed effects on episodic memory, results from the second experiment confirm the dissociation of attention and memory for everyday objects found in the first experiment. A limitation of the study consists in the relatively small sample sizes. Future studies should replicate the results and could also extend this approach to other memory paradigms, such as encoding of verbal material and intentional encoding designs.

In summary, our results demonstrate that episodic memory encoding of everyday objects is enhanced by appetitive and aversive associations acquired through a lifetime of experience with such objects. These memory effects are found after short retention intervals and can be dissociated from the effects of motivation on attention. Effects of visual contrast confirm this dissociation of attention and memory, suggesting that even during a free-viewing task, memory encoding can occur independently of overt attention.

## Author Contributions

BCW and JS: experimental design and writing; JS: data analysis.

## Conflict of Interest Statement

The authors declare that the research was conducted in the absence of any commercial or financial relationships that could be construed as a potential conflict of interest.

## Supplementary Material

Individual participant data are available at http://dx.doi.org/10.5281/zenodo.802156
