# Supplementary material for: Memory Performance for Everyday Motivational and Neutral Objects Is Dissociable from Attention
Source: Front Behav Neurosci. 2017 Jun 26;11:121. doi: 10.3389/fnbeh.2017.00121 (PMC5483478; doi:10.3389/fnbeh.2017.00121)
Supplement: Supplementary file 1 [file Data_Sheet_1.docx]

Supplementary Material

**Memory Performance for Everyday Motivational and Neutral Objects is Dissociable from Attention**Schomaker, J*, & Wittmann, B.C.
*****Corresponding Author: judith.schomaker@gmail.com

# Supplementary Tables

For completeness, we here report generalized logistic model results for models for which memory success was defined as both correct remember and know responses (i.e. hits) for old objects without familiarization. The main findings as found for recollection are also found for overall recognition memory: Motivation predicted memory success in experiment 1, and visual contrast predicted memory success in experiment 2.

*Table S1. Generalized logistic mixed model results on remember and know responses to old objects without familiarization, separately for experiment*

|  | Experiment 1 | Experiment 2 |
| --- | --- | --- |
| *Observations* | 1026 for 19 subjects | 918 for 17 subjects |
| Motivation | **E=-0.20, SE=0.15, *z*=-1.31, *p*=.019** | E=-0.25, SE=0.17, *z*=-1.42, *p*=.154 |
| Quadratic Motivation | **E=0.20, SE=0.09, *z*=2.31, *p*=.021** | E=-0.17, SE=0.19, *z*=-0.92, *p*=.356 |
| Valence | E=0.40, SE=0.21, *z*=1.92, *p*=.054 | E=0.38, SE=0.21, *z*=1.82, *p*=.070 |
| Quadratic Valence | E=0.17, SE=0.10, *z*=1.74, *p*=.081 | E=-0.28, SE=0.34.*z* = -0.82, *p*=.415 |
| Arousal | E=0.11, SE=0.17, *z*=0.65, *p*=.516 | E=0.40, SE=0.23, *z*=1.78, *p*=.075 |
| Recognizability | E=0.17, SE=0.14, *z*=1.19, *p*=.233 | E=0.17, SE=0.14, *z*=1.23, *p*=.220 |
| Total viewing duration | E=-0.13, SE=.28, *z*=-0.47, *p*=.639 | E=0.54, SE=0.35, *z*=1.53, *p*=.126 |
| Visual contrast |  | **E=2.11, SE=0.65, *z*=3.25, *p*=.001** |
| Quadratic Visual contrast |  | **E=-0.54, SE=0.16, *z*=-3.36, *p*<.001** |

*Significant effects (p < .05) are printed in bold. E = Estimate; SE = standard error*

*Table S2 Generalized logistic mixed model results on remember and know responses to old objects without familiarization including first fixation latency as a predictor, separately for experiment 1 and 2.*

|  | Experiment 1 | Experiment 2 |
| --- | --- | --- |
| *Observations* | 1026 for 19 subjects | 918 for 17 subjects |
| Motivation | E=-0.17, SE=0.16, *z*=-1.09, *p*=.274 | E=-0.24, SE=0.17, *z*=-1.37, *p*=.171 |
| Quadratic Motivation | **E=0.21, SE=0.09, *z*=2.27, *p*=.023** | E=-0.15, SE=0.19, *z*=-0.80, *p*=.422 |
| Valence | E=0.39, SE=0.22, *z*=1.83, *p*=.068 | E=0.38, SE=0.21, *z*=1.79, *p*=.074 |
| Quadratic Valence | E=0.14, SE=0.10, *z*=1.34, *p*=.180 | E=-0.31, SE=0.34.*z* = -0.92, *p*=.359 |
| Arousal | E=0.15, SE=0.19, *z*=0.82, *p*=.412 | E=0.41, SE=0.23, *z*=1.85, *p*=.064 |
| Recognizability | E=0.20, SE=0.15, *z*=1.31, *p*=.189 | E=0.16, SE=0.14, *z*=1.11, *p*=.269 |
| First Fixation Latency | E<0.01, SE<.01, *z*=-0.67, *p*=.502 | E<-0.01, SE<0.01, *z*=0.60, *p*=.549 |
| Visual contrast |  | **E=2.06, SE=0.65, *z*=3.17, *p*=.001** |
| Quadratic Visual contrast |  | **E=-0.52, SE=0.16, *z*=-3.25, *p*=.001** |

*Significant effects (p < .05) are printed in bold. E = Estimate; SE = standard error*

*Table S3 Generalized logistic mixed model results on remember and know responses to old objects without familiarization including mean novel fixation duration as a predictor, separately for experiment 1 and 2.*

|  | Experiment 1 | Experiment 2 |
| --- | --- | --- |
| *Observations* | 1026 for 19 subjects | 918 for 17 subjects |
| Motivation | E=-0.09, SE=0.16, *z*=-0.55, *p*=.580 | E=-0.30, SE=0.17, *z*=-1.71, *p*=.087 |
| Quadratic Motivation | **E=0.25, SE=0.09, *z*=2.74, *p*=.006** | E=-0.15, SE=0.19, *z*=-0.79, *p*=.431 |
| Valence | E=0.30, SE=0.21, *z*=1.39, *p*=.164 | **E=0.45, SE=0.21, *z*=2.10, *p*=.036** |
| Quadratic Valence | E=0.11, SE=0.09, *z*=1.10, *p*=.271 | E=-0.36, SE=0.34.*z* = -1.06, *p*=.289 |
| Arousal | E=0.13, SE=0.18, *z*=0.69, *p*=.492 | E=0.40, SE=0.23, *z*=1.76, *p*=.079 |
| Recognizability | E=0.08, SE=0.15, *z*=0.57, *p*=.567 | E=0.18, SE=0.14, *z*=1.22, *p*=.223 |
| Mean Novel Fixation | E<-0.01, SE<.01, *z*=-1.18, *p*=.238 | E=-0.00, SE=0.00, *z*=-1.22, *p*=.224 |
| Visual contrast |  | **E=2.34, SE=0.66, *z*=3.56, *p*<.001** |
| Quadratic Visual contrast |  | **E=-0.59, SE=0.16, *z*=-3.65, *p*<.001** |

*Significant effects (p < .05) are printed in bold. E = Estimate; SE = standard error*
